# First Isolation and Rapid Identification of Newcastle Disease Virus from Aborted Fetus of Dromedary Camel Using Next-Generation Sequencing

**DOI:** 10.3390/v11090810

**Published:** 2019-09-01

**Authors:** Jade Lee Lee Teng, Ulrich Wernery, Hwei Huih Lee, Sunitha Joseph, Joshua Fung, Shyna Korah Elizabeth, Kai Yan Yeong, Joerg Kinne, Kwok-Hung Chan, Susanna Kar Pui Lau, Patrick Chiu Yat Woo

**Affiliations:** 1 Department of Microbiology, The University of Hong Kong, Hong Kong, China; 2 State Key Laboratory of Emerging Infectious Diseases, The University of Hong Kong, Hong Kong, China; 3 Carol Yu Centre for Infection, The University of Hong Kong, Hong Kong, China; 4 Central Veterinary Research Laboratory, Dubai 00000, UAE; 5 Collaborative Innovation Center for Diagnosis and Treatment of Infectious Diseases, The University of Hong Kong, Hong Kong, China

**Keywords:** Newcastle Disease virus, aborted fetus, dromedary

## Abstract

Newcastle disease virus (NDV) causes morbidities and mortalities in wild and domestic birds globally. For humans, exposure to infected birds can cause conjunctivitis and influenza-like symptoms. NDV infections in mammals are rarely reported. In this study, using next-generation sequencing, an NDV was identified and isolated from Vero cells inoculated with the nasal swab of an aborted dromedary fetus in Dubai, during the time when an NDV outbreak occurred in a pigeon farm located in close proximity to the dairy camel farm where the mother of the aborted dromedary fetus resided, and there were a lot of pigeons in the camel farm. Genome analysis revealed that the structurally and functionally important features of other NDVs were also present in this dromedary NDV genome. Phylogenetic analysis based on the nucleotide sequences of fusion protein (F), hemagglutinin-neuraminidase protein (HN) and complete polyprotein showed that the virus belonged to sub-genotype VIg of class II NDV and is most closely related to pigeon NDVs in Egypt in the same year. The present study is the first that demonstrated isolation of NDV in dromedaries. Further study is warranted to investigate the relationship between NDV infection and abortion.

## 1. Introduction

Newcastle disease virus (NDV), synonymous with *Avian avulavirus 1*, is a negative-sense single-stranded RNA virus that belongs to the genus *Avulavirus* of the *Paramyxoviridae* family. It is an avian virus causing high morbidities and mortalities in most species of wild and domestic birds globally and is associated with major economic loss in poultry farms worldwide [1]. Transmission of the virus occurs through exposure to the fecal materials and other excretions of infected birds or contaminated food, water and equipment [1,2,3,4]. In birds, NDV mainly causes diseases in the respiratory, neurological, and gastrointestinal systems. Occasionally, NDV has also caused infections in mammals, such as cattle, sheep, and mink [5,6,7]. In 1952, NDV was isolated from the lung of a six-month-old calf with pneumonia in the United States [7]. In 2012, it was isolated from the blood of two apparently healthy sheep in India [6]. In 2017, it was isolated from an outbreak of hemorrhagic encephalitis and pneumonia in domestic mink from mainland China [5]. For humans, exposure to infected birds can cause mild self-limiting conjunctivitis and influenza-like symptoms.

The NDV genome comprises a single-stranded negative sense RNA that encodes for a nucleocapsid protein (N), a phosphoprotein (P), a matrix protein (M), a fusion protein (F), a hemagglutinin-neuraminidase protein (HN), and a large polymerase protein (L), from the 3′ terminus to the 5′ terminus. Based on the complete coding sequence of the F protein [8,9,10,11], NDV strains are classified into class I and class II, with class II NDVs being more diverse, and are further classified into genotypes I to XVIII. In terms of pathogenicity, NDVs can be categorized into velogenic (virulent), mesogenic (intermediate), or lentogenic (non-virulent), which have been linked to their virulence in chickens or the presence of conserved amino acids in the F and HN proteins which are known to be involved in pathogenesis of NDV infection, such as virus attachment, host-cell membrane fusion, virus dissemination, and F protein activation [12].

Although NDV is known to infect some mammalian species, it has never been isolated or amplified from camels. However, a previous study reported that a pigeon NDV isolate was able to agglutinate camel red blood cells, achieving haemagglutination titer comparable to the level obtained with chicken red blood cells [13]. This suggested that haemagglutination protein binding receptor was also present on the membrane of camel red blood cells, implicating that camel may be susceptible to NDV infection [13]. In this article, we report the first isolation of NDV from the aborted fetus of a dromedary in the United Arab Emirates during the process of Middle East Respiratory Syndrome coronavirus (MERS-CoV) screening, and results of its comparative genome and phylogenetic analysis.

## 2. Materials and Methods

### 2.1. Sample Collection and Viral Culture

Clinical sample of an aborted dromedary fetus was obtained during necropsy at the Central Veterinary Research Laboratory in Dubai, the United Arab Emirates, using the standard procedures. Nasal swab was initially inoculated onto Vero cells for MERS-CoV screening but the RT-PCR result was negative [14]. Viral transport medium, which contained Modified Hank’s Balanced Salt Solution supplemented with 5% of bovine serum albumin, 20% of glucose, amphotericin B (4 µg/mL), colistin (7.5 µg/mL), and vancomycin (100 µg/mL), was used to dilute the clinical sample 10-fold and filtered. Two hundred microliters of the filtrate was mixed with 200 μL of minimum essential medium (Gibco, Thermo Fisher Scientific, Waltham, MA, USA), and the mixture was further inoculated into 24-well tissue culture plate with Vero cells to allow adsorption for 1 h. The excess inoculum was discarded, phosphate-buffered saline was used to wash the wells twice, and the medium was replaced with 1 mL of minimum essential medium (Gibco, USA). Culture was incubated at 37 °C with 5% CO_2_ and inspected daily for cytopathic effects (CPE) by inverted microscopy.

Two hundred microliters of the cell culture supernatant sample were collected and inoculated into 24-well tissue culture plate with chicken embryo fibroblasts. Culture was incubated at 37 °C with 5% CO_2_ and examined daily for the appearance of CPE.

### 2.2. Hemagglutination (HA) Test

To test for the presence of haemagglutinin, 25 μL of PBS was mixed with 25 μL of the cell culture isolate and the mixture was added to the well of a V-shaped microtitre plate according to procedures described in the OIE Terrestrial Manual 2018 [15]. Next, 25 μL of 1% suspension of chicken red blood cells was added followed by incubation at room temperature for 30 min. The cell culture isolate was tested in triplicate together with controls. Clumping of the red blood cells (i.e., agglutinated cells settled as a diffuse film) is considered as positive for the presence of haemagglutinin. HA titer of the virus is defined as the maximum dilution showing visible agglutination and expressed as haemagglutinating units (HAU)/mL.

### 2.3. Preparation of Sample for Illumina Sequencing

The QIAamp Viral RNA Mini Kit (Qiagen, Hilden, Germany) was used to extract viral RNA from the culture supernatants of infected Vero cells. Reverse transcription and PCR were performed using SuperScript III reverse transcriptase (Invitrogen, Carlsbad, CA, USA) using primer contained a 20-base arbitrary sequence at the 5′ end followed by a randomized octamer (8 N) at the 3′ end as described previously [16]. A single round priming and extension was performed using Klenow fragment polymerase (New England Biolabs, Ipswich, MA, USA). PCR amplification were performed in an automated thermal cycler (Applied Biosystems, Foster City, CA, USA) with primer consisting of only the 20-based arbitrary sequence of the random primer with 20 cycles of 94 °C for 15 s, 60 °C for 30 s and 68 °C for 1 min and a final extension of 68 °C for 7 min. Standard precautions were taken to avoid PCR contamination and no amplified PCR product was observed in negative control. The PCR product was purified following the manufacturer’s protocol using the MinElute PCR Purification Kit (Qiagen, Germany) with slight modification. The purified DNA was eluted in 15 μL of EB buffer and used as the template for library construction.

### 2.4. Library Construction for Illumina Sequencing

The DNA library was prepared using Nextera XT DNA sample preparation Kit (Illumina, San Diego, CA, USA) according to the manufacturer’s protocol and using strategies as described previously [16]. First, 1 ηg of DNA was incubated with the Nextera XT transposome at 55 °C for 5 min. The transposome fragmented the input DNA and added adapter sequence to the ends to allow PCR amplification in downstream procedures. Next, the library with tagmented DNA was amplified for 12 cycles of 95 °C for 10 s, 55 °C for 30 s and 72 °C for 30 s and a final extension of 72 °C for 5 min. Amplified DNA library was purified using AMPure XP beads (1.8×) (Beckman Coulter, Brea, CA, USA) to remove short fragments from the population. The 2100 Bioanalyzer instrument (Agilent Technologies, Santa Clara, CA, USA) and KAPA library quantification kit (Kapa Biosystems, Wilmington, MA, USA) were used to analyze and quantify the library, respectively. The verified library was sequenced on Illumina HiSeq 1500 (151 bp paired-end with Rapid Run Mode). Image analysis and base calling were performed using SCS2.8/RTA1.8 (Illumina, USA) and FASTQ file generation and the removal of failed reads were generated using CASAVA ver.1.8.2 (Illumina, USA).

### 2.5. Analysis of Sequence Reads and Genome Assembly

Illumina sequence raw reads were trimmed using Trimmomatic-0.36 with parameters of leading 3, trailing 3, sliding window 4:15, minimum length 36 bp [17]. Trimmed paired-end reads that were aligned to bacterial genomes, host genomes, and rRNA sequences from the SILVA rRNA database were removed using the DeconSeq 0.4.3 software [18]. CD-HIT v4.6.4 was used to cluster the filtered reads that are similar [19]. BLASTx with an E-value cutoff of 10^−5^ were used to align the clustered reads towards non-redundant (nr) viral protein sequences from NCBI database [20]. The remaining reads were compared to nr protein sequence database using BLASTx with the same E-value cutoff. The final BLAST output was analyzed by matching each sequence to its best hit. The taxonomical content of the dataset was generated and visualized by MEtaGenome ANalyzer (MEGAN) version 6.6.7 where phylogenetic tree computed was assigned to each sequence according to its taxonomical identity in NCBI database [21].

Since reads related to NDV were found abundantly in the phylogenetic tree by MEGAN analysis, corresponding sequenced reads of 453,417 were extracted, yielding an average NDV genome coverage of >4000×. The extracted paired-end reads were *de novo* assembled into contigs using accurate mode with MIRA version 4.9.6 [22]. The assembled contigs were subject to additional genome analysis by comparing with their corresponding closest relatives.

### 2.6. Comparative Genome Analysis and Phylogenetic Analysis

The ORF Finder [23] was used to predict the putative open reading frames (ORFs) and the deduced amino acid sequences. The nucleotide sequences of the genomes and the deduced amino acid sequences of the ORFs were compared to those of other known viruses using MUSCLE by multiple sequence alignment [24]. Phylogenetic analysis based on nucleotide sequence was performed using MEGA-X [25], with bootstrap values calculated from 500 trees. Phylogenetic trees were constructed using maximum-likelihood according to the best-fit model (Kimura-2 + G + I) determined by MEGA-X [25]. All genotypes of NDVs were included in the phylogenetic analysis.

### 2.7. Genome Sequencing of Newcastle Disease Virus (NDV) Isolated from Dromedary Camel (DcNDV)

Genome of DcNDV was amplified and sequenced using previously published strategies [26]. Original culture isolate was used to extract the viral RNA using QIAamp Viral RNA mini kit (Qiagen, Hilden, Germany). The SuperScript III kit (Invitrogen, Carlsbad, CA, USA) was used for reverse transcription. PCR primers were designed based on the genome sequences assembled using HiSeq data (Table S1). Sequences were assembled and edited manually using BioEdit 7.2.6.1 [27] to produce the final sequences of the viral genomes.

## 3. Results

### 3.1. Necropsy

Necropsy of the fresh female 75-cm long fetus with greyish edematous placenta revealed swollen body lymph nodes, excessive bloody fluid in thoracic and abdominal cavities, non-ventilated lung, and brownish fluid in the gastrointestinal tract. Histopathology showed marked edema of the organs and diffuse mineralization of the placenta with no inflammation. Moreover, no microorganism or viral inclusions were observed.

### 3.2. Virus Culture and Haemagglutination (HA) Test

**Figure 1:**
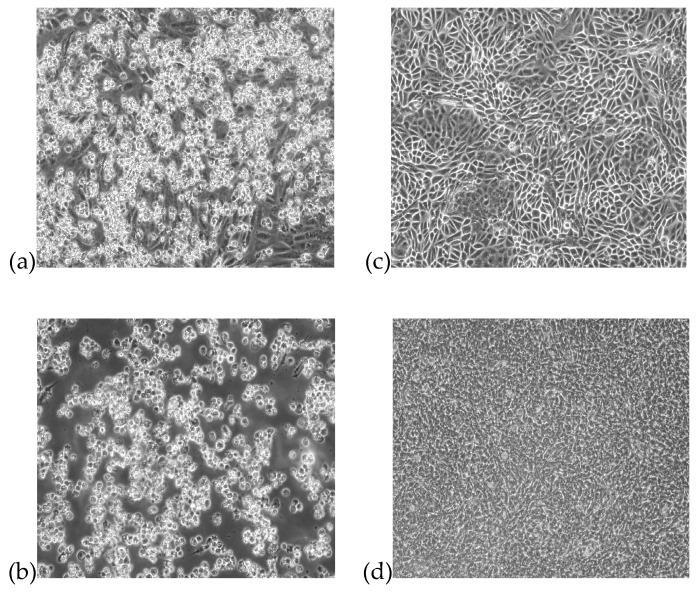
Cytopathic effects of dromedary camel Newcastle disease virus (DcNDV) culture isolate from the aborted fetus of a dromedary on (**a**) Vero cells; (**b**) chicken embryo fibroblasts, showing cell rounding, degeneration and detachment. (**c**) Uninfected control of Vero cells; (**d**) Uninfected control of chicken embryo fibroblasts. Magnification: 100×.

At the first passage, the Vero cells inoculated with the nasal swab sample showed CPE on day 3 with cell rounding, progressive degeneration, and detachment (Figure 1a). Further passage of the virus in chicken embryo fibroblasts also showed CPE with cell rounding and detachment (Figure 1b). The corresponding mock-infected controls were shown in Figure 1c,d. The virus was able to agglutinate 1% of chicken red blood cells and the HA titer was 640 HAU/mL.

### 3.3. Deep Sequence Analysis

The unknown culture isolate from the nasal swab of the diseased camel fetus was deep sequenced using the HiSeq 1500 instrument, generating 28,366,102 pair-end 151-bp reads. Ribosomal RNA sequences, bacterial and host genomes were filtered from the trimmed paired-end reads, resulting in 17,483,318 clean reads which were used for downstream BLASTx analysis. Among these clean reads, 454,406 reads matched to viruses. The largest portion of these viral sequences (*n* = 453,656, 99.8%) were assigned to the family *Paramyxoviridae*, with the majority of reads (*n* = 453,417, 99.0%) matched to NDVs.

### 3.4. Genome Analysis

The DcNDV genome was assembled from the metagenomics data and most of the genome sequence was further confirmed by RT-PCR and DNA sequencing, except the 5′- and 3′- untranslated regions. The DcNDV genome was identical to the genome sequence assembled from metagenomics data. The size of the polyprotein gene of DcNDV was 13,746 bases and the G + C content was 46%. The genome organization was consistent with other NDVs, with the characteristic gene order 3′-N-P-M-F-HN-L-5′.

Detail annotation of the complete coding region of the F (3996–5657 nt, 1662 bp, 554 aa) and HN (5864–7579 nt, 1713 bp, 571 aa) genes of the DcNDV revealed structurally and functionally important residues and motifs which are commonly observed in other NDVs (Table 1, Table 2 and Table 3, Figures S1 and S2). These conserved residues and motifs in the F protein included the fusion peptide (117–142 aa), heptad repeat region-a (143–185 aa), heptad repeat region-b (268–299 aa), heptad repeat region-c (471–500 aa), major transmembrane domain (501–521 aa), five predicted *N*-glycosylation sites at ^85^NRT^87^, ^191^NNT^193^, ^366^NTS^368^, ^447^NIS^449^, and ^471^NNS^499^ and 13 cysteine residues at positions 25, 27, 76, 199, 338, 347, 362, 370, 394, 399, 401, 424, and 523 (Table 1, Figure S1) [28,29]. In addition, the F protein also contained the cleavage site motif ^112^KRQKRF^117^, which is typical of velogenic NDV strains (Table 1, Figure S1) [28,29]. As for the HN protein, the DcNDV contained transmembrane domain at position 25–45, stalk and head domains at position 54–123 and 124–571, six predicted *N*-glycosylation sites at^119^NNS^121^, ^341^NNT^343^, ^433^NKT^435^, ^481^NHT^483^, ^508^NIS^510^, and ^538^NKT^540^, thirteen cysteine residues at positions 123, 172, 187, 196, 238, 247, 251, 344, 455, 461, 465, 531, and 542 and heptad repeat region at position 74–88 and 96–110 (Table 2 and Table 3, Figure S2) [28,30]. Moreover, the HN protein contained the conserved amino acid residues at neutralizing epitopes, including site 23 (193–211 aa) and site 1 and site 14 (345–355 aa), site 12 (494 aa), site 2 and site 12 (513–521 aa), and site 2 (569 aa). These features and its protein size (i.e., 571 aa) are also typical of velogenic NDV strains (Table 2 and Table 3, Figure S2) [30]. Overall, the DcNDV possessed important residues and motifs that are more closely related to those of NDV strains of genotype VI (Table 1, Table 2 and Table 3).

### 3.5. Phylogenetic Analysis

**Figure 2:**
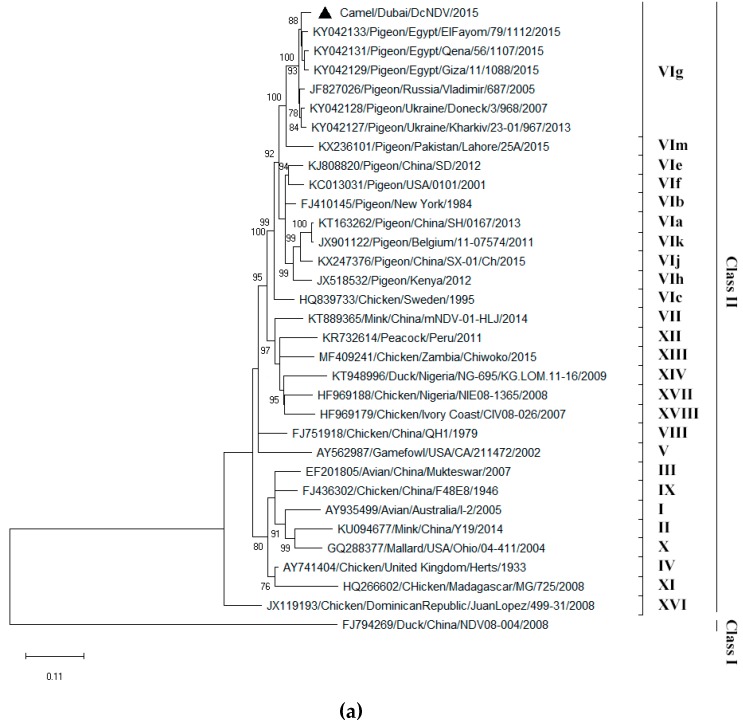

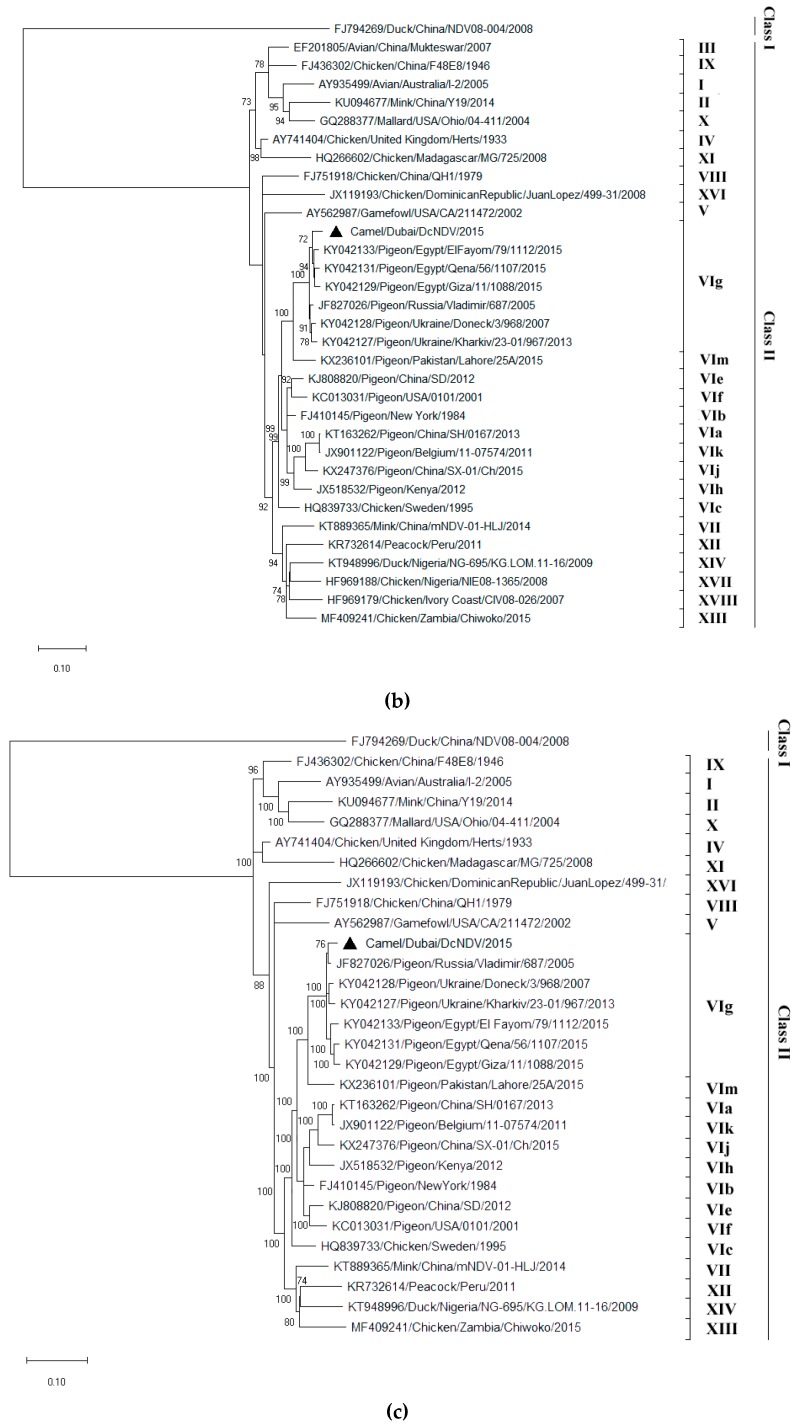
Phylogenetic analyses of the complete nucleotide sequences of (**a**) fusion protein (F) gene; (**b**) hemagglutinin-neuraminidase (HN) gene; (**c**) concatenated genes of dromedary camel Newcastle disease virus (DcNDV) discovered in the present study. A total of 1662, 1716, 13,745 nucleotide positions of F gene, HN gene, and concatenated genes were included in the analyses respectively. Bootstrap values below 70% are not shown. The scale bar indicates the number of nucleotide substitutions per site. The DcNDV detected in this study is marked with a solid black triangular ( ). All the accession numbers are given as cited in GenBank.

To determine the phylogenetic relationship between the DcNDV and other NDV strains reported, phylogenetic trees based on the nucleotide sequences of F, HN, and complete polyprotein of DcNDV and those of other NDVs were constructed (Figure 2a–c). Phylogenetic analysis of the complete F gene sequences revealed that the DcNDV was most closely related to members of class II genotype VI NDVs (Figure 2a). Within genotype VI, it was most closely related to members of sub-genotype g, sharing the highest nucleotide identity (95%) to strain Pigeon/Egypt/ElFayom/79/1112/2015 (GenBank accession number KY042133). Consistently, phylogenetic analyses based on the HN and the complete polyprotein sequence displayed similar topologies (Figure 2a–c). The pairwise nucleotide identities of all the coding genes of DcNDV and representative strains from Class II genotype VI were shown in Table 4. Sequence analysis based on the complete polyprotein sequences showed that DcNDV shared the highest nucleotide identities, ranging from 96.8 to 97.8%, to the NDVs found in the pigeons from Egypt (Genbank accession numbers KY042129, KY042131, and KY042133), Russia (Genbank accession number JF827026) and Ukraine (Genbank accession numbers KY042128 and KY042127). There were only 18 and 22 amino acid differences between the F and HN proteins of DcNDV and those of the three closest pigeon NDV strains from Egypt, respectively (Figures S1 and S2). Detailed examination of the amino acid alignments revealed that the amino acid changes in the regions containing structurally and functionally important residues and motifs were all conservative substitutions (Figures S1 and S2).

### 3.6. Nucleotide Sequence GenBank Accession Number

The nucleotide sequence of the genome of the DcNDV in this study has been lodged within the GenBank sequence database under accession no. MK673997. Raw data of Illumina HiSeq have been submitted to Sequence Read Archive (SRA) under accession no. SRR8569164.

## 4. Discussion

We report the first isolation of NDV in a dromedary. NDV is basically an avian virus which has caused numerous outbreaks of devastating disease in poultries. In the present study, we isolated an NDV from the nasal swab of an aborted dromedary fetus in Dubai using Vero cells, and the virus isolate also produced the typical CPE caused by NDV on chicken embryo fibroblasts. During that period, an NDV outbreak occurred in the pigeon farm located in close proximity to the dairy camel farm where the mother of the aborted dromedary fetus resided and there were a lot of pigeons in the camel farm. We speculate that the mother dromedary had probably acquired the NDV from the pigeons and subsequently transmitted to the fetus. Whole genome sequencing and comparative genome analysis revealed that the structurally and functionally important features in the F and HN proteins of the NDV genome observed in previous studies were also present in the genome of this NDV in dromedary. In particular, it also possessed genomic features similar to other velogenic NDVs, including cleavage site motif ^112^KRQKRF^117^ in the F protein, conserved amino acid residues at neutralizing epitopes on the HN protein, and the size (i.e., 571 aa) of the HN protein (Table 1, Table 2 and Table 3, Figures S1 and S2) [30,31]. On the other hand, we were aware of the limitation of the present study. Viral cultures on other samples of the fetus or the mother were not performed because it was initially intended for MERS-CoV isolation. It is noteworthy that a virus that could be successfully cultured and isolated from Vero cells was subjected to investigation by next-generation sequencing. This suggested that any potential pathogens that were not able to propagate in the Vero cells may have been missed. Nevertheless, although the possibility of NDV infection being responsible for abortion is unknown, the successful isolation of NDV from the aborted dromedary fetus and the absence of positive bacterial and fungal cultures in the placental samples warrant further investigations on the causal relationship between NDV infection and abortion.

The present dromedary NDV was probably resulted from spillover from NDVs in birds of the Middle East. NDVs of class II genotype VI were first isolated from pigeons in the Middle East in the 1960s and spread rapidly throughout Northern Africa to Europe and other parts of the world [11,32]. So far, NDVs of genotype VI have been identified in at least 20 countries of three different continents, including Africa, Europe, and Asia [10,32]. In addition to their widespread distribution, genotype VI NDVs is also highly diverse genetically, dividing into sub-genotypes VIa to Vim [8,9,10,11,32]. In 2015, NDVs of sub-genotype VIg were detected in apparently healthy pigeons kept in captivity in Egypt [32]. Phylogenetic analysis showed that the NDV isolated from the aborted dromedary fetus in the present study belongs to sub-genotype VIg and is most closely related to the NDVs found in these healthy pigeons from Egypt in the same year (Figure 2. In fact, whole genome comparative analysis revealed that there is only 2.5–5.3% difference in the nucleotide sequence between the dromedary NDV from Dubai and the pigeon NDVs from Egypt, substantiating the theory that the virus probably had transmitted directly from pigeons to dromedary. Notably, they also shared almost the same conserved residues and domains responsible for virulence in the F and HN proteins, suggesting that the present dromedary NDV may also be velogenic, similar to other velogenic NDVs of genotype VI (Figures S1 and S2) [12,33]. Moreover, the present findings are in line with a spillover event recently reported in Nigeria, in which a high degree of sequence similarity between NDVs from domestic and wild birds was observed [34]. The study also discovered some bird species that had not previously been known to be infected by NDVs [34]. Although it is known that pigeons are not migratory, the mechanism of spread of genotype VI NDVs at long distances (i.e., from Egypt to Dubai) in the present study remains to be determined. A possible explanation for the spread of genotype VI NDVs may be due to the international trade of racing pigeons [33]. Further studies to determine the genotype of NDVs circulating in pigeons in Dubai or other parts of the Middle East and to screen for additional NDVs in camels are warranted to understand the present spillover event and the evolution of NDVs.

The use of next generation sequencing technologies has led to the discovery of many novel viruses as well as identification of known viruses that were previously not reported in dromedaries. The MERS epidemic and discovery of dromedaries as the reservoir of MERS-CoV and our discovery and isolation of a novel dromedary camel CoV UAE-HKU23 have boosted interest in search of more novel viruses in dromedaries [35,36,37]. In metagenomics studies and subsequent whole genome sequencing, we discovered two novel genotypes of hepatitis E virus (HEV), a novel genus of enterovirus, a novel astrovirus, two novel bocaparvoviruses, and novel picobirnaviruses and circoviruses in dromedaries [26,38,39,40,41,42,43,44,45]. In addition to these metagenomics studies, we have also used next generation sequencing technologies in a previous study and the present study to rapidly identify and sequence the genomes of a West Nile virus and an NDV, respectively [46]. These are the first studies that demonstrate isolation of West Nile virus and NDV in dromedaries. All these studies have remarkably widened the spectrum of viruses found in dromedaries as well as the potential source of infections due the corresponding viruses.

## Figures and Tables

**Table 1:** Comparison of amino acids in functional domains of the fusion (F) protein of dromedary camel Newcastle disease virus (DcNDV) with corresponding sequences of representative NDV strains from Class II genotype.

Strains/Genotypes	Cleavage Site Motif	Fusion Peptide	Heptad Repeats			Cysteine Residues	Transmembrane Domain
			HRa	HRb	HRc		
	112–117	117–142	143–185	268–299	471–500		501–521
DcNDV	KRQKRF	FIGAIIGSVALGVATSAQITAAAAL	QANQNAANILRLKESIAATNEAVHEVTDGLSQLAVAVGKMQQF	LYDSQTQLLGIQVNLPSVGNLNNMR	NNSISNALDRLAESNSKLDKVNVKLTSTSA	25, 27, 76, 199, 338, 347, 362, 370, 394, 399, 401, 424, 523	LITYIILTVISLIFGALSLIL
Avian/Australia/I-2/2005/I	RKQGRL	LIGAIIGGVALGVATAAQITAASAL	QANQNAANILRLKESIAATNEAVHEVTDGLSQLAVAVGKMQQF	LYDSQTQLLGIQVILPSVGNLNNMR	NNSISNALDKLEESNSKLDKVNVKLASTSA	25, 27, 76, 199, 338, 347, 362, 370, 394, 399, 401, 424, 523	LITYIVLTVISLICGILSLVL
Mink/China/Y19/2014/II	GRQGRL	LIGAIIGGVALGVATAAQITAAAAL	QAKQNAANILRLKESIAATNEAVHEVTDGLSQLAVAVGKMQQF	LYDSQTQLLGIQVTLPSVGNLNNMR	NNSTSNALNKLEESNSKLDKVNVKLTSTSA	25, 27, 76, 199, 338, 347, 362, 370, 394, 399, 401, 424, 523	LITYIVLTIISLVFGILSLVL
Avian/China/Mukteswar/2007/III	RRQRRF	FIGAIIGGVALGVATAAQITAASAL	QANQNAANILRLKESIAATNEAVHEVTGGLSQLAVAVGKMQQF	FYDSQTQLLGIQVTLPSVGNLNNMR	NHSISNALDKLEESNSKLDKVNVRLTSTSA	25, 27, 76, 199, 338, 347, 362, 370, 394, 399, 401, 424, 523	LITYIVLTVISLVLGMLSLVL
Chicken/UnitedKingdom/Herts/1933/IV	RRQRRF	FIGAIIGGVALGVATAAQITAASAL	QANQNAANILRLKESIAATNEAVHEVTDGLSQLAVAVGKMQQF	LYDSQTQILGIQVTLPSVGNLNNMR	NNSISNALNKLEESNSKLDKVNVRLTSTSA	25, 27, 76, 199, 338, 347, 362, 370, 394, 399, 401, 424, 523	LITYIVLTVISLVFGVLSLVL
Gamefowl/USA/CA/211472/2002/V	RRQKRF	FVGAIIGGVALGVATAAQITAAAAL	QANQNAANILRLKESIAATNDAVHEVTNGLSQLAVAVGKMQQF	LYDSQTQLLGIQINLPSVGSLNNMR	NNSISSTLDKLAESNNKLNKVNVNLTSTSA	25, 27, 76, 199, 338, 347, 362, 370, 394, 399, 401, 424, 523	LITYIVLAIVSLAFGVISLVL
Pigeon/Egypt/ElFayom/79/1112/2015/VI	KRQKRF	FIGAIIGSVALGVATSAQITAAAAL	QANQNAANILRLKESIAATNEAVHEVTDGLSQLAVAVGKMQQF	LYDSQTQLLGIQVNLPSVGNLNNMR	NNSISNALDRLAESNSKLDKVNVKLTSTSA	25, 27, 76, 199, 338, 347, 362, 370, 394, 399, 401, 424, 523	LITYIVLTVISLVFGALSLIL
Mink/China/mNDV-01-HLJ/2014/VII	RRQKRF	FIGAIIGSVALGVATAAQITAAAAL	QANQNAANILRLKESIAATNEAVHEVTDGLSQLSVAVGKMQQF	LYDSQTQLLGIQVNLPSVGNLNNMR	NNSISNALDKLAESNSKLEKVNVRLTSTSA	25, 27, 76, 199, 338, 347, 362, 370, 394, 399, 401, 424, 523	LITYIVLTVISLVFGALSLGL
Chicken/China/QH1/1979/VIII	RRQKRF	FIGAVIGSVALGVATAAQITAAAAL	QANQNAANILRLKESIAATNEAVHEVTDGLSQLSVAVGKMQQF	LYDSQTQLLGIQVTLPSVGNLNNMR	NNSISNALDKLSESNSKLDKVNVKLTSTSA	25, 27, 76, 199, 338, 347, 362, 370, 394, 399, 401, 424, 523	LIIYIVLIVISLVSGVLSLVL
Chicken/China/F48E8/1946/IX	RRQRRF	FIGAVIGSVALGVATAAQITAASAL	QANQNAANILRLKESIAATNEAVHEVTDGLSQLSVAVGKMQQF	LYDSQTQLLGIQVTLPSVGNLNNMR	NNSISNALDKLEESNSKLDKVNVKLTGTSA	25, 27, 76, 199, 338, 347, 362, 370, 394, 399, 401, 424, 523	LITYIVLTIISLVCGILSLVL
Mallard/USA/Ohio/04-411/2004/X	EKQGRL	LIGAIIGGVALGVATAAQITAASAL	QANQNAANILRLKESIAATNEAVHEVTDGLSQLSVAVGKMQQF	LYDSQTQLLGIQVTLPSVGNLNNMR	NNSISNALDKLEESNSKLDKVNVKLTSTSA	25, 27, 76, 199, 338, 347, 362, 370, 394, 399, 401, 424, 523	LITYIILTVVSLVCGILSLVL
Chicken/Madagascar/MG/725/2008/XI	RRRRRF	FVGAIIGSVALGVATAAQITAAAAL	QAKQNAANILRLKESIAATNEAVHEVTDGLSQLSVAVGKMQQF	LYDSQTQILGIQVTMPSVGSLNNMR	NNSISKTLNKLEESNSKLDRVNVKLASTSA	25, 27, 76, 199, 338, 347, 362, 370, 394, 399, 401, 424, 523	LITYIVLTVVSLVFGVLSLVL
Peacock/Peru/2011/XII	RRQKRF	FIGAVIGSVALGVATAAQITAAAAL	QANQNAANILRLKESIAATNEAVHEVTDGLSQLSVAVGKMQQF	LYDSQTQLLGIQVNLPSVGNLNNMR	NNSISNALDKLAESNSKLDKINVRLTSTSA	25, 27, 76, 199, 338, 347, 362, 370, 394, 399, 401, 424, 523	LITYIVLTAISLVFGTLSLVL
Chicken/Zambia/Chiwoko/2015/XIII	RRQKRF	FIGAVIGSVALGVATAAQITAAAAL	QANQNAANILRLKESIAATNEAVHEVTDGLSQLSVAVGKMQQF	LYDSQTQLLGIQVNLPSVGNLNNMR	NNSISNALDKLAESNNKLDKVNVRLTSTSA	25, 27, 76, 199, 338, 347, 362, 370, 394, 399, 401, 424, 523	LITYIVLTVTSLVFGALSLAL
Duck/Nigeria/NG-695/KG.LOM.11-16/2009/XIV	RRRKRF	FVGAVIGSVALGVATAAQVTAAAAL	QANQNAANILRLKESIAATNEAVHEVTDGLSQLSVAVGKMQQF	LYDPQTQLLGIQVNLPSVGNLNNMR	NNSISNALDKLAESNNKLDKVNVRLTSASA	25, 27, 76, 199, 338, 347, 362, 370, 394, 399, 401, 424, 523	LITYIVLTGLSLMFGTLSLVL
Chicken/DominicanReplubic/JuanLopez/499-31/2008/XVI	RRQKRF	FVGAIIGGVALGVATAAQITAAAAL	QANQNAANILRLKESIAATNEAVHEVTDGLSQLSVAVGKMQQF	LYDPQTQLLGIQVTLPSVGNLNNMR	NNSISNALSKLAESNSKLSKVNVKLTSTSA	25, 27, 76, 199, 338, 347, 362, 370, 394, 399, 401, 424, 523	LITYIVLTVVSLAFGVISLVL
Chicken/Nigeria/NIE08-1365/2008/XVII	RRQKRF	FIGAVIGSVALGVATAAQITAAAAL	QANQNAANILRLKESIAATNEAVHEVTDGLSQLSVAVGKMQQF	LYDSQTQLLGIQVSLPSVGNLNNMR	NNSISSALDKLAESNSKLDKVNVRLTSTSA	25, 27, 76, 199, 338, 347, 362, 370, 394, 399, 401, 424, 523	LITYIVLTVVSLVFGTLSLVL
Chicken/IvoryCoast/CIV08-026/2007/XVIII	RRQKRF	FVGAVIGSVALGVATAAQITAAAAL	QANQNAANILRLKESIAATNEAVHEVTDGLSQLSVAVGKMQQF	LYDSQTQLLGIQVSLPSVGNLNNMR	NNSISNALDKLAESNSKLDKVNVRLTSTSA	25, 27, 76, 199, 338, 347, 362, 370, 394, 399, 401, 424, 523	LITYIVLTVISLVFGTLSLIL

**Table 2:** Comparison of amino acids in functional domains of the hemagglutinin-neuraminidase (HN) protein of dromedary camel Newcastle disease virus (DcNDV) with corresponding sequences of representative NDV strains from Class II genotype.

Strains/Genotypes	Transmembrane Domain	Heptad Repeats		Receptor Recognition	N-linked Glycosylation Sites	Cysteine Residues
		HRa	HRb			
	25–45	74–88	96–110	174, 175, 198, 236, 258, 299, 317, 401, 416, 498, 526, 547		
DcNDV	FRIAVLFLVVMTLAISAAVL	LGSNQDVVDRIYKQV	LLNTESVIMNAITSL	R, I, D, K, E, Y, Y, E, R, R, Y, E	119, 341, 433, 481, 508, 538	123, 172, 186, 196, 238, 247, 251, 344, 455, 461, 465, 531, 542
Avian/Australia/I-2/2005/I	FRIAILFLTVVTLAVSAAAL	LGSNQDVVDRIYKQV	LLNTESTIMNAITSL	R, I, D, K, E, Y, Y, E, R, R, Y, E	119, 341, 433, 481, 538	123, 172, 186, 196, 238, 247, 251, 344, 455, 461, 465, 531, 542
Mink/China/Y19/2014/II	FRIAILLLTIVTLAISVISL	LGSNQDVVDRIYKQV	LLSTETTIMNAITSL	R, I, D, K, E, Y, Y, E, R, R, Y, E	119, 341, 433, 481, 538	172, 186, 196, 238, 247, 251, 344, 455, 461, 465, 531, 542
Avian/China/Mukteswar/2007/III	FRIAALLLMVITLAVSAVAL	LGSNQDVVDRIYKQV	LLNTESIIMNAITSL	R, I, D, K, E, Y, Y, E, R, R, Y, E	119, 341, 433, 481, 508, 538	123, 172, 186, 196, 238, 247, 251, 344, 455, 461, 465, 531, 542
Chicken/UnitedKingdom/Herts/1933/IV	FRIAILLLIVITLAISAAAL	LSSNQDVVDRIYKQV	LLNTESVIMNAITSL	R, I, D, K, E, Y, Y, E, R, R, Y, E	119, 341, 433, 481, 508, 538	123, 172, 186, 196, 238, 247, 251, 344, 455, 461, 465, 531, 542
Gamefowl/USA/CA/211472/2002/V	FRVAVLSLIVMTLAISVAAL	LNSNQDVVDRVYKQV	LLNTESIIMNAITSL	R, I, D, K, E, Y, Y, E, R, R, Y, E	119, 341, 433, 481, 538	123, 172, 186, 196, 238, 247, 251, 344, 455, 461, 465, 531, 542
Pigeon/Egypt/ElFayom/79/1112/2015/VI	FRITVLLLVVMTLAISAAVL	LGSNQDVVDRIYKQ	LLNTESIIMNAITSL	R, I, D, K, E, Y, Y, E, R, R, Y, E	119, 341, 433, 481, 508, 538	123, 172, 186, 196, 238, 247, 251, 344, 455, 461, 465, 531, 542
Mink/China/mNDV-01-HLJ/2014/VII	FRIAVLLLMVMTLAISAAAL	LSSSQDVIDRIYKQV	LLNTKSIIMNAITSL	R, I, D, K, E, Y, Y, E, R, R, Y, E	119, 341, 433, 481, 508, 538	123, 172, 186, 196, 238, 247, 251, 344, 455, 461, 465, 531, 542
Chicken/China/QH1/1979/VIII	FRIAVLFLIVTTLAISAAAL	LSSNQDVVDRIYKQV	LLNTESIIMNAITSL	R, I, D, K, E, Y, Y, E, R, R, Y, E	119, 341, 433, 481, 508, 538	123, 172, 186, 196, 238, 247, 251, 344, 455, 461, 465, 531, 542
Chicken/China/F48E8/1946/IX	FRTAVILLIVVTFSISAAAL	LGSNQDVVDRIYKQV	LLNTESIIMSAITSL	R, I, D, K, E, Y, Y, E, R, R, Y, E	119, 341, 433, 481, 508, 538	123, 172, 186, 196, 238, 247, 251, 344, 455, 461, 465, 531, 542
Mallard/USA/Ohio/04-411/2004/X	FRIAILLLTVVTLAISAAAL	LGSNQDVVDRIYKQV	LLNTESTIMNAITSL	R, I, D, K, E, Y, Y, E, R, R, Y, E	119, 341, 433, 481, 538	123, 172, 186, 196, 238, 247, 251, 344, 455, 461, 465, 531, 542
Chicken/Madagascar/MG/725/2008/XI	FRIAILLLITITLALSTAAL	LSSNRDVMDRVYKQV	LLSTESVIMNAITSL	R, I, D, K, E, Y, Y, E, R, R, Y, E	119, 341, 433, 481, 508, 538	123, 172, 186, 196, 238, 247, 251, 344, 455, 461, 465, 531, 542
Peacock/Peru/2011/XII	FRVSVLLLMVMTLAISAVAL	LSSSQDVIDRIYKQV	LLNTESIIMNAITSL	R, I, D, K, E, Y, Y, E, R, R, Y, E	119, 341, 433, 481, 538	123, 172, 186, 196, 238, 247, 251, 344, 455, 461, 465, 531, 542
Chicken/Zambia/Chiwoko/2015/XIII	FRTAVLLLIVMTLAISIAAL	LSSSQDVVDRIYKQV	LLNTESVIMNAITSL	R, I, D, K, E, Y, Y, E, R, R, Y, E	119, 341, 433, 481, 538	123, 172, 186, 196, 238, 247, 251, 344, 455, 461, 465, 531, 542
Duck/Nigeria/NG-695/KG.LOM.11-16/2009/XIV	FRIAVLLLMVMTLAISAAAL	LGSSQDVIDRIYKQV	LLNTESIIMNAITSL	R, I, D, K, E, Y, Y, E, R, R, Y, E	119, 341, 433, 481, 508, 538	123, 172, 186, 196, 238, 247, 251, 344, 455, 461, 465, 531, 542
Chicken/DominicanReplubic/JuanLopez/499-31/2008/XVI	FRITVLLFILMTLAISVATL	LGSNQDVVDRIYKQV	LLNTESIIMNALTSL	R, I, D, K, E, Y, Y, E, R, R, Y, E	119, 341, 433, 481, 508, 538	123, 172, 186, 196, 238, 247, 251, 344, 455, 461, 465, 531, 542
Chicken/Nigeria/NIE08-1365/2008/XVII	FRVAVLLLIAVTLAVSAAAL	LSSSQDVIDRIYKQV	LLNTESILMNAITSL	R, I, D, K, E, Y, Y, E, R, R, Y, E	119, 341, 433, 481, 508, 538	123, 172, 186, 196, 238, 247, 251, 344, 455, 461, 465, 531, 542
Chicken/IvoryCoast/CIV08-026/2007/XVIII	FRIAVLFLMTMTFVISAAAL	LSSSQDVIDRIYKQV	LLNTESIIMNAITSL	R, I, D, K, E, Y, Y, E, R, R, Y, E	119, 341, 433, 481, 508, 538	123, 172, 186, 196, 238, 247, 251, 344, 455, 461, 465, 531, 542

**Table 3:** Comparison of amino acids residues of the neutralizing epitopes and C-terminal domain of the hemagglutinin-neuraminidase (HN) protein of dromedary camel Newcastle disease virus (DcNDV) with corresponding sequences of representative NDV strains from Class II genotype.

Strains/Genotypes	Neutralizing Epitopes		C-terminal Domains		
	193–211	345–355	494	513–521	569
DcNDV	LSGCRDHSHSHQYLALGV	PDEQSYQIRMA	D	RVTRVSSGS	D
Avian/Australia/I-2/2005/I	LSGCRDHSHSHQYLALGV	PDEQDYQIRMA	D	RITRVSSGS	D
Mink/China/Y19/2014/II	LSGCRDHSHSHQYLALGV	PDEQDYQIRMA	G	RITRVSSSS	D
Avian/China/Mukteswar/2007/III	LSGCRDHTHSHQYLALGV	PDEQDYQIRMA	D	RITRVSSSS	G
Chicken/UnitedKingdom/Herts/1933/IV	LSGCRDHSHSHQYLALGV	PDEQDYQIRMA	D	RITRVSSRS	D
Gamefowl/USA/CA/211472/2002/V	LSGCRDHSHSHQYLALGV	PDEQDYQVRMA	N	RITRVSSTS	D
Pigeon/Egypt/ElFayom/79/1112/2015/VI	LSGCRDHSHSHQYLALGV	PDEQSYQIRMA	D	RVTRVSSGS	D
Mink/China/mNDV-01-HLJ/2014/VII	LSGCRDHSHSHQYLALGV	PDEQDYQIRMA	D	RVTRVSSSS	D
Chicken/China/QH1/1979/VIII	LSGCRDHSHSHQYLALGV	PDEQDYQIRMA	D	RMTRVSSSS	D
Chicken/China/F48E8/1946/IX	LSGCRDHSHSHQYLALGV	PDEQDYQIRMA	D	RITRVSSSS	D
Mallard/USA/Ohio/04-411/2004/X	LSGCRDHSHSYQYLALGV	PDEQDYQIRMA	D	RITRVSSSS	D
Chicken/Madagascar/MG/725/2008/XI	LSGCRDHSHSHQYLALGV	PDEQDYQIRMA	D	RITRVSSSS	G
Peacock/Peru/2011/XII	LSGCRDHSHSHQYLALGV	PDEQDYQIRMA	G	RVTRVSSSS	D
Chicken/Zambia/Chiwoko/2015/XIII	LSGCRDHSHSHQYLALGV	PDEQDYQIRMA	D	RVTRVSSSS	A
Duck/Nigeria/NG-695/KG.LOM.11-16/2009/XIV	LSGCRDHSHSHQYLALGV	PDEQDYQIRMA	D	RVTRVSSSS	D
Chicken/DominicanReplubic/JuanLopez/499-31/2008/XVI	LSGCRDHSHSHQYLALGV	PDEQDYQIRMA	D	RITRVSSSS	D
Chicken/Nigeria/NIE08-1365/2008/XVII	LSGCRDHSHSHQYLALGV	PDEQDHQIRMA	D	HVTRVSSSS	D
Chicken/IvoryCoast/CIV08-026/2007/XVIII	LSGCRDHSHSHQYLALGV	PDEQDYQIRMA	D	RVTRVSSSS	D

**Table 4:** Comparison of nucleotide sequences of dromedary camel Newcastle disease virus (DcNDV) with corresponding sequences of representative NDV strains from Class II genotype VI.

Reference Strains	Accession	Genotype Nucleotide Differences (%) with DcNDV							
	Number		N	P	M	F	HN	L	Concatenated Genes
Pigeon/China/SH/0167/2013	KT163262	VIa	90.7	86.5	90.3	90.4	88.1	91.4	90.3
Pigeon/USA/Maryland/1984	FJ410147	VIb	92.9	89.2	92.5	92.2	91.1	93.3	92.4
Crested ibis/China/Shaanxi10/2010	KC853020	VIc	90.5	87.7	90.0	91.0	89.0	90.9	90.3
Pigeon/China/SD/2012	KJ808820	VIe	90.6	89.3	91.2	91.0	90.2	92.2	91.3
Pigeon/USA/PA/0712/2007	KC013040	VIf	90.7	87.7	90.2	91.6	89.3	91.4	90.7
Pigeon/Egypt/El_Fayom/79/1112/2015	KY042133	VIg	96.4	95.9	97.5	97.0	96.7	97.1	96.9
Pigeon/Egypt/Qena/56/1107/2015	KY042131	VIg	96.5	95.3	97.0	97.1	96.4	97.2	96.8
Pigeon/Egypt/Giza/11/1088/2015	KY042129	VIg	97.0	94.7	96.9	97.1	96.3	97.2	96.8
Pigeon/Russia/Vladimir/687/2005	JF827026	VIg	97.3	96.5	97.4	96.9	96.8	98.8	97.8
Pigeon/Ukraine/Doneck/3/968/2007	KY042128	VIg	97.0	95.9	96.9	96.8	96.2	97.6	97.1
Pigeon/Ukraine/Kharkiv/23-01/967/2013	KY042127	VIg	96.7	95.4	96.5	96.5	96.0	97.3	96.8
Pigeon/Kenya/2012	JX518532	VIh	86.7	86.3	90.1	90.6	88.8	91.4	89.9
Pigeon/China/SX-01/Ch/2015	KX247376	VIj	91.2	87.7	91.0	90.3	88.1	91.0	90.3
Pigeon/Belgium/11-07574/2011	JX901122	VIk	90.6	86.7	90.6	90.4	88.1	91.4	90.3

## Supplementary Materials

The following are available online at https://www.mdpi.com/1999-4915/11/9/810/s1, Table S1: Primers used for genome sequencing of DcNDV, Figure S1, Alignment of deduced amino acid sequence of complete F gene of DcNDV, Figure S2, Alignment of deduced amino acid sequence of complete HN gene of DcNDV.
