# Supplementary material for: First Isolation and Rapid Identification of Newcastle Disease Virus from Aborted Fetus of Dromedary Camel Using Next-Generation Sequencing
Source: Viruses. 2019 Sep 1;11(9):810. doi: 10.3390/v11090810 (PMC6783818; doi:10.3390/v11090810)
Supplement: Supplementary file 1 [file viruses-11-00810-s001.pdf]

Table S1. Primers used for genome sequencing of DcNDV.

Primer pairs	Primers name	Location	Primers (5′ – 3′)
**1**	LPW 38283	1 -800	ATGTCATCCGTCTTTGAC
	LPW 38284		AATGCAGTTAACCCGGTG
**2**	LPW 38285	700 – 1,500	GAGCTTAAGAGGGGACGC
	LPW 38286		GCCCGGTGGGGGCGGGCT
**3**	LPW 38287	1,396 – 2,293	CAGAGCACTACTCATCTGG
	LPW 38288		GCTTCCAGGGGCGGCTTT
**4**	LPW 38289	2,192 – 2,950	GAAAACCACCATCAACCC
	LPW 38290		CAGCGCAAGGCGTTTGAT
**5**	LPW 38291	2,854 – 3,674	ACGCCCGATGCATCCAAA
	LPW 38292		CCGCTCCCAGGGATCTTC
**6**	LPW 38293	3,558 – 4,377	CTTCTCAGTAATGCAGGC
	LPW 38294		TTCTAATTAGATAGACAG
**7**	LPW 36838	4,681 – 5,762	CGGCAGTGTAGCTCTTGGGG
	LPW 36839		CCGCTAACCTATCCAGGGCA
**8**	LPW 38295	4,261 – 5,085	AAGAAGTAAGCTGCACCC
	LPW 38296		CGTAGTCAATTCAGTTAG
**9**	LPW 38297	4,969 – 5,806	AAGATGCAGCAGTTTGTT
	LPW 38298		TGCCCGTCACGATGACTT
**10**	LPW 38299	5,699 – 6,507	GATAGATAGACATTCATG
	LPW 38300		TAATCTTCTCCTCCGCCT
**11**	LPW 37470	6,368 – 7,216	AGAACACATGGCGTTTGGTT
	LPW 37471		AAGGATCCCGTTCCCACT
**12**	LPW 38301	6,389 – 7,222	TCTAGTAGTAATGACTTT
	LPW 38302		TCAATAAAGGATCCGCTT
**13**	LPW 37466	6,430 – 6,977	TGCATAGCATGAGGGCCAG
	LPW 37467		GATGGAACGCAGAGTCGAG
**14**	LPW 38303	7,103 – 7,937	AATGGTGCACGGAAGGTT
	LPW 38304		TTCTGCAATGCTAAGACA
**15**	LPW 38305	7,817 – 8,657	CAACATATCCCGCAGTCG
	LPW 38306		AACTTTGCGAACAGGTCT
**16**	LPW 38307	8,535 – 9,369	CCATCCCAGGTGTTTAGA
	LPW 38308		GCGAGACTCAAGCAGTGG
**17**	LPW 38309	9,138 – 10,072	ATATAGCAGAATCAGTAA
	LPW 38310		ATAGTTGACTCATCGCTA
**18**	LPW 38311	9,953 – 10,780	ACTGCCAAGTAATGGCAG
	LPW 38312		TAAGATCGCCTGATATTA
**19**	LPW 38313	10,661 – 11,506	GTGAAACCATCAGGTCAG
	LPW 38314		TCCGTGTCAGCCTCTTGA
**20**	LPW 38315	11,370 – 12,190	ACGTGTCGCGCATGCTAT
	LPW 38316		CCTCTTTGACTCCTTCTT
**21**	LPW 38317	12,074 – 12,901	TAGATGATGGCATAACAC
	LPW 38318		CAGACAATTCGATAAAAT
**22**	LPW 38319	12,777 – 13,602	TCTACTCTCTAATATTGC
	LPW 38320		TTCCGCCAAGTATAAGGA
**23**	LPW 38321	13, 478 – 14,290	ACCTAGTACGGTATTTGT
	LPW 38322		CTATTACACGATGCTGTT
**24**	LPW 38323	14,172 – 14,880	TGTGCACGAGGTGGTGAG
	LPW 38324		TTAAGAGTCATTGTCACA

Figure S1. Alignment of deduced amino acid sequence of complete F gene of DcNDV. The residue profile of DcNDV is compared with the three closest pigeon NDV strains from Egypt. Structurally and functionally important domains are indicated. Amino acid changes are highlighted in yellow. Cleavage site motif is bolded in red font, fusion peptide is shaded in grey, heptad repeats are in orange font, cysteine residues are shown with arrows and transmembrane domain is in purple font.

```
Camel/Dubai/DcNDV_F/2015                     MSSKSSSRTPAPLMLITRIMLILSCICLTSSLDGRPLAAAGIVVTGDKAVNIYTSSQTGS   60
KY042133/Pigeon/Egypt/ElFayom/79/112/2015    MSSKSSTRIPAPLMLITRIMLILSCICLTSSLDGRPLAAAGIVVTGDKAVNIYTSSQTGS   60
KY042131/Pigeon/Egypt/Qena/56/1107/2015      MSSKSSTRIPAPLMLITRIMLILSCICLTSSLDGRPLAAAGIVITGDKAVNIYTSSQTGS   60
KY042129/Pigeon/Egypt/Giza/11/1088/2015      MSSKSSTRIPAPLMLITRIMLILSCICPTSPLDGRPLAAAGIVITGDKAVNIYTSSQTGS   60
                                             ******:* ****************** ** ************:****************

Camel/Dubai/DcNDV_F/2015                     IIIKLLPNMPKDKEACARAPLEAYNRTLTALLNPLGDSIRRIQGSVSTSGGKRQKRFIGA   120
KY042133/Pigeon/Egypt/ElFayom/79/112/2015    IIIKLLPNMPKDKEACARAPLEAYNRTLTALLNPLGDSIRRIQGSVSTSGGKRQKRFIGA   120
KY042131/Pigeon/Egypt/Qena/56/1107/2015      IIIKLLPNMPKDKEACARAPLEAYNRTLTALLNPLGDSIRRIQGSVSTSGGKRQKRFIGA   120
KY042129/Pigeon/Egypt/Giza/11/1088/2015      IIIKLLPNMPKDKEACARAPLEAYNRTLTALLNPLGDSIRRIQGSVSTSGGKRQKRFIGA   120
                                             ************************************************************

Camel/Dubai/DcNDV_F/2015                     IIGSVALGVATSAQITAAAALIQANQNAANILRLKESIAATNEAVHEVTDGLSQLAVAVG   180
KY042133/Pigeon/Egypt/ElFayom/79/112/2015    IIGSVALGVATSAQITAAAALIQANQNAANILRLKESIAATNEAVHEVTDGLSQLAVAVG   180
KY042131/Pigeon/Egypt/Qena/56/1107/2015      IIGSVALGVATSAQITAAAALIQANQNAANILRLKESIAATNEAVHEVTDGLSQLAVAVG   180
KY042129/Pigeon/Egypt/Giza/11/1088/2015      IIGSVALGVATSAQITAAAALIQANQNAANILRLKESIAATNEAVHEVTDGLSQLAVAVG   180
                                             ************************************************************

Camel/Dubai/DcNDV_F/2015                     KMQQFVNDQFNNTARELDCIKITQQVGIELNLYLTELTTVFGPQITSPALTRLTIQALYN   240
KY042133/Pigeon/Egypt/ElFayom/79/112/2015    KMQQFVNDQFNNTARELDCIKVTQQVGIELNLYLTELTTVFGPQITSPALTQLTIQALYN   240
KY042131/Pigeon/Egypt/Qena/56/1107/2015      KMQQFVNDQFNNTARELDCIKVTQQVGIELNLYLTELTTVFGPQITSPALTQLTIQALYN   240
KY042129/Pigeon/Egypt/Giza/11/1088/2015      KMQQFVNDQFNNTARELDCIKVTQQVGIELNLYLTELTTVFGPQITSPALTQLTIQALYN   240
                                             *********************:*****************************:********

Camel/Dubai/DcNDV_F/2015                     LAGGNMDYLLTKLGIGNNQLSSLIGSGLITGHPILYDSQTQLLGIQVNLPSVGNLNNMRA   300
KY042133/Pigeon/Egypt/ElFayom/79/112/2015    LAGGNMDYLLTKLGIGNNQLSSLIGSGLITGHPILYDSQTQLLGIQVNLPSVGNLNNMRA   300
KY042131/Pigeon/Egypt/Qena/56/1107/2015      LAGGNMDYLLTKLGIGNNQLSSLIGSGLITGHPILYDSQTQLLGIQVNLPSVGNLNNMRA   300
KY042129/Pigeon/Egypt/Giza/11/1088/2015      LAGGNMDYLLTKLGIGNNQLSSLIGSGLITGHPILYDSQTQLLGIQVNLPSVGNLNNMRA   300
                                             ************************************************************

Camel/Dubai/DcNDV_F/2015                     TYLGTLSVSTTKGFASALVPKVVTQVGSVIEELDTSHCIESDLDLYCTRIVTFPMSPSIY   360
KY042133/Pigeon/Egypt/ElFayom/79/112/2015    TYLGTLSVSTTKGFASALVPKVVTQVGSVIEELDTSHCIESDLDLYCTRIVTFPMSPGIY   360
KY042131/Pigeon/Egypt/Qena/56/1107/2015      TYLGTLSVSTTKGFASALVPKVVTQVGSVIEELDTSHCIESDLDLYCTRIVTFPMSPGIY   360
KY042129/Pigeon/Egypt/Giza/11/1088/2015      TYLGTLSVSTTKGFASALVPKVVTQVGSVIEELDTSHCIESDLDLYCTRIVTFPMSPGIY   360
                                             *********************************************************.**

Camel/Dubai/DcNDV_F/2015                     SCLSGNTSACMYSKTEGALTTPYMALKGSVIANCKITTCRCVDPPGIISQNYGEAVSLID   420
KY042133/Pigeon/Egypt/ElFayom/79/112/2015    SCLSGNTSACMYSKTEGALTTPYMALKGSVIANCKITTCRCVDPPGIISQNYGEAVSLID   420
KY042131/Pigeon/Egypt/Qena/56/1107/2015      SCLSGNTSACMYSKTEGALTTPYMALKGSVIANCKITTCRCVDPPGIISQNYGEAVSLID   420
KY042129/Pigeon/Egypt/Giza/11/1088/2015      SCLSGNTSACMYSKTEGALTTPYMALKGSVIANCKITTCRCIDPPGIISQNYGEAVSMID   420
                                             *****************************************:***************:**

Camel/Dubai/DcNDV_F/2015                     RHSCNVISLDGITLRLSGEFDVTYQKNISILDSQVIVTGNLDISTELGNVNNSISNALDR   480
KY042133/Pigeon/Egypt/ElFayom/79/112/2015    RHSCKILSLDGITLRLSGEFDVTYQKNISILDSQVIVTGNLDISTELGNVNNSISNALDR   480
KY042131/Pigeon/Egypt/Qena/56/1107/2015      RHSCNVLSLDGITLRLSGEFDVTYQKNISILDSQVIVTGNLDISTELGNVNNSISNALDR   480
KY042129/Pigeon/Egypt/Giza/11/1088/2015      RHSCNVLSLDGITLRLSGEFDVTYQKNISILDSQVIVTGNLDISTELGNVNNSISNALDR   480
                                             ****:::*****************************************************

Camel/Dubai/DcNDV_F/2015                     LAESNSKLDKVNVKLTSTSALITYIILTVISLIFGALSLILACYLMYKQKAQQKTLIGLG   540
KY042133/Pigeon/Egypt/ElFayom/79/112/2015    LAESNSKLDKVNVKLTSTSALITYIVLTVISLVFGALSLILACYLMYKQKAQQKTLIWLG   540
KY042131/Pigeon/Egypt/Qena/56/1107/2015      LAESNSKLDKVNVKLTSTSALITYIVLTVISLVFGALSLILACYLMYKQKAQQKTLIWLG   540
KY042129/Pigeon/Egypt/Giza/11/1088/2015      LAESNSKLDKVNVKLTSTSALITYIVLTVISLVFGALSLILACYLMYKQKAQQKTLIWLG   540
                                             *************************:******:************************ **

Camel/Dubai/DcNDV_F/2015                     NNTLDQMRATTRT      553
KY042133/Pigeon/Egypt/ElFayom/79/112/2015    NNTLDQMRATTRT      553
KY042131/Pigeon/Egypt/Qena/56/1107/2015      NNTLDQMRATART      553
KY042129/Pigeon/Egypt/Giza/11/1088/2015      NNTLDQMRATTRT      553
                                             **********:**
```

Figure S2. Alignment of deduced amino acid sequence of complete HN gene of DcNDV. The residue profile of DcNDV is compared with the three closest pigeon NDV strains from Egypt. Structurally and functionally important domains are indicated. Amino acid changes are highlighted in yellow. Transmembrane domain is in green font, heptad repeats are in orange font, receptor recognition sites are in blue font, neutralizing epitopes are shaded in grey, C-terminal domains are in red font, n-linked glycosylation sites are bolded and cysteine residues are shown with arrows.

```
Camel/Dubai/DcNDV_HN/2015                   MDQAVRRVVLENEEREAKNTWRLVFRIAVLFLVVMTLAISAAVLVHSMRASTPRDIVSIS   60
KY042133/Pigeon/Egypt/ElFayom/79/112/2015   MDRAVRRVVLENEEREAKNTWRLVFRITVLLLVVMTLAISAAVLVHSMRASTPRDIVSIS   60
KY042131/Pigeon/Egypt/Qena/56/1107/2015     MDRAVRRVVLENEEREAKNTWRLVFRIVVLLLVVMTLAISAAVLVHSMRASTPRDIVSIS   60
KY042129/Pigeon/Egypt/Giza/11/1088/2015     MDRAVRRVVLENEEREAKNTWRLVFRIAVLLLVVMTLAISAAVLVHSMRASTPRDIVSVS   60
                                            **:************************.**:***************************:*

Camel/Dubai/DcNDV_HN/2015                   TVISKAEEKITSLLGSNQDVVDRIYKQVALESPLALLNTESVIMNAITSLSHQINGAANN   120
KY042133/Pigeon/Egypt/ElFayom/79/112/2015   TVISKTEEKITSLLGSNQDVVDRIYKQVALESPLALLNTESIIMNAITSLSHQINGAANN   120
KY042131/Pigeon/Egypt/Qena/56/1107/2015     TVISKTEEKITSLLGSNQDVVDRIYKQVALESPLALLNTESIIMNAITSLSHQINGAANN   120
KY042129/Pigeon/Egypt/Giza/11/1088/2015     TVISKTEEKITSLLSSNQDVVDRIYKQVALESPLALLNTESIIMNAITSLSHQINGAANN   120
                                            *****:********.**************************:******************

                                              ▼                                                ▼
Camel/Dubai/DcNDV_HN/2015                   SGCGAPVHDPDYIGGIGKELIVDDTSDVTSFYPSSYQEHLNFIPAPTTGSGCTRIPSFDM   180
KY042133/Pigeon/Egypt/ElFayom/79/112/2015   SGCGAPVHDPDYIGGIGKELIVDDTSDVTSFYPSAYQEHLNFIPAPTTGSGCTRIPSFDM   180
KY042131/Pigeon/Egypt/Qena/56/1107/2015     SGCGAPVHDPDYIGGIGKELIVDDTSDVTSFYPSAYQEHLNFIPAPTTGSGCTRIPSFDM   180
KY042129/Pigeon/Egypt/Giza/11/1088/2015     SGCGAPVHDPDYIGGIGKELIVDDTSDVTSFYPSAYQEHLNFIPAPTTGSGCTRIPSFDM   180
                                            **********************************:*************************

                                                 ▼         ▼                                         ▼
Camel/Dubai/DcNDV_HN/2015                   SATHYCYTHNVILSGCRDHSHSHQYLALGVLRTSATGRVFFSTLRSINLDDTQNRKSCSV   240
KY042133/Pigeon/Egypt/ElFayom/79/112/2015   SATHYCYTHNVILSGCRDHSHSHQYLALGVLRTSATGRVFFSTLRSINLDDAQNRKSCSV   240
KY042131/Pigeon/Egypt/Qena/56/1107/2015     SATHYCYTHNVILSGCRDHSHSHQYLALGVLRTSATGRVFFSTLRSINLDDAQNRKSCSV   240
KY042129/Pigeon/Egypt/Giza/11/1088/2015     SATHYCYTHNVILSGCRDHSHSHQYLALGVLRTSATGRVFFSTLRSINLDDAQNRKSCSV   240
                                            ***************************************************:********

                                                  ▼   ▼
Camel/Dubai/DcNDV_HN/2015                   SATPLGCDILCSKVTETEEEDYKSITPTSMVHGRLGFDGRYHEKDLDITVLFKDWVANYP   300
KY042133/Pigeon/Egypt/ElFayom/79/112/2015   SATPLGCDILCSKVTETEEEDYKSIAPTPMVHGRLGFDGQYHEKDLDITVLFKDWVANYP   300
KY042131/Pigeon/Egypt/Qena/56/1107/2015     SATPLGCDILCSKVTETEEEDYKSIAPTSMVHGRLGFDGQYHEKDLDITVLFKDWVANYP   300
KY042129/Pigeon/Egypt/Giza/11/1088/2015     SATPLGCDILCSKVTETEEEDYKSIAPTSMVHGRLGFDGQYHEKDLDITVLFKDWVANYP   300
                                            *************************:** **********:********************

Camel/Dubai/DcNDV_HN/2015                   GVGSGSFIDDRVWFPVYGGLKPNSPSDTAQEGNYAIYKRYNNTCPDEQSYQIRMAKASYK   360
KY042133/Pigeon/Egypt/ElFayom/79/112/2015   GVGSGSFIDDRVWFPVYGGLKPNSPSDTAQEGNYVIYKRYNNTCPDEQSYQIRMAKASYK   360
KY042131/Pigeon/Egypt/Qena/56/1107/2015     GVGSGSFIDDRVWFPVYGGLKPNSPSDNAQEGNYVIYKRYNNTCPDEQNYQIRMAKASYK   360
KY042129/Pigeon/Egypt/Giza/11/1088/2015     GVGSGSFIDDRVWFPVYGGLKPNSPSDTAQEGNYVIYKRYNNTCPDEQSYQIRMAKASYK   360
                                            ***************************.******.*************.***********

Camel/Dubai/DcNDV_HN/2015                   PGRFGGKRVQQAILSIKVSTSLGEDPVLTIPPNTITLMGAEGRILTVGTSHFLYQRGSSY   420
KY042133/Pigeon/Egypt/ElFayom/79/112/2015   PGRFGGKRVQQAILSIKVSTSLGEDPVLTVPPNTITLMGAEGRILTVGTSHFLYQRGSSY   420
KY042131/Pigeon/Egypt/Qena/56/1107/2015     PGRFGGKRVQQAILSIKVSTSLGEDPVLTVPPNTITLMGAEGRILTVGTSHFLYQRGSSY   420
KY042129/Pigeon/Egypt/Giza/11/1088/2015     PGRFGGKRVQQAILSIKVSTSLGEDPVLTVPPNTITLMGAEGRILTVGTSHFLYQRGSSY   420
                                            *****************************:******************************

                                                                              ▼     ▼   ▼
Camel/Dubai/DcNDV_HN/2015                   FSPALLYPMTVYNKTATLHSPYTFNAFTRPGIVPCQASARCPNSCITGVYTDPYPLIFHR   480
KY042133/Pigeon/Egypt/ElFayom/79/112/2015   FSPALLYPLTVHNKTATLHSPYTFNAFTRPGIVPCQASARCPNSCITGVYTDPYPLIFYR   480
KY042131/Pigeon/Egypt/Qena/56/1107/2015     FSPALLYPMTVHNKTATLHSPYTFNAFTRPGIVPCQASARCPNSCITGVYTDPYPLIFHR   480
KY042129/Pigeon/Egypt/Giza/11/1088/2015     FSPALLYPMTVHNRTATLHSPYTFNAFTRPGIVPCQASARCPNSCITGVYTDPYPLIFYR   480
                                            ********:**:*:********************************************:*

Camel/Dubai/DcNDV_HN/2015                   NHTLRGVFGTMLDDIQARLNPVSAVFDNISRSRVTRVSSGSTKAAYTTSTCFKVVKTNKT   540
KY042133/Pigeon/Egypt/ElFayom/79/112/2015   NHTLRGVFGTMLDDIQARLNPVSAVFDNISRSRVTRVSSGSTKAAYTTSTCFKVVKTNKT   540
KY042131/Pigeon/Egypt/Qena/56/1107/2015     NHTLRGVFGTMLDDIQARLNPVSAVFDNISRSRVTRVSSGSTKAAYTTSTCFKVVKTNKT   540
KY042129/Pigeon/Egypt/Giza/11/1088/2015     NHTLRGVFGTMLDDIQARLNPVSAVFDNTSRSRVTRVSSGSTKAAYTTSTCFKVVKTNKT   540
                                            **************************** *******************************

                                             ▼
Camel/Dubai/DcNDV_HN/2015                   YCLSIAEISNTLFGEFRIVPLLVEILKGDRA           571
KY042133/Pigeon/Egypt/ElFayom/79/112/2015   YCLSIAEISNTLFGEFRIVPLLVEILKGDRV           571
KY042131/Pigeon/Egypt/Qena/56/1107/2015     YCLSIAEISNTLFGEFRIVPLLVEILKGDRV           571
KY042129/Pigeon/Egypt/Giza/11/1088/2015     YCLSIAEISNTLFGEFRIVPLLVEILKGDRV           571
                                            ******************************
```
